# Prevalence and Patterns of Patient-Reported Missed or Delayed Injuries in Polytrauma Patients and Their Association With Health-Related Quality of Life Using the 36-Item Short Form Health Survey

**DOI:** 10.7759/cureus.109697

**Published:** 2026-05-26

**Authors:** Ganesh Gude, Atif Saeed, Ayesha Mansoor, Zeeshan Umar, Rena Yousuf, Shijas Shanavas, Shivam Singla, Bhavna Singla, Sunita Kumawat, Shaumile H Khan, Syed Laveeza

**Affiliations:** 1 Medicine, Mamata General Hospital, Khammam, IND; 2 Emergency Medicine, University of Health Sciences Lahore, Lahore, PAK; 3 Special Pathology/Community Medicine/ENT/Opthalmology, Allied Hospital Faisalabad, Faisalabad Medical University, Faisalabad, PAK; 4 Emergency Medicine, Pak Emirates Military Hospital (PEMH), Rawalpindi, PAK; 5 Medicine, Ras Al-Khaimah Medical and Health Sciences University, Ras Al-Khaimah, ARE; 6 Medicine, Ibrahim Bin Hamad Obaidullah Hospital, Ras Al-Khaimah, ARE; 7 Internal Medicine, TidalHealth Peninsula Regional, Salisbury, USA; 8 Internal Medicine, Erie County Medical Center, Buffalo, USA; 9 Internal Medicine, St. Francis Medical Center, Lynwood, USA; 10 Medicine, Fatima Memorial Hospital College of Medicine and Dentistry, Lahore, PAK; 11 Medicine, Islamic International Medical College (IIMC), Riphah International University, Rawalpindi, PAK

**Keywords:** missed injuries, polytrauma, quality of life, sf-36, study-specific patient-reported missed injuries questionnaire

## Abstract

Purpose

Polytrauma patients may feel that certain injuries were not identified during the initial examination. The current study analyzed the prevalence of patient-reported missed or delayed injuries and their correlation with health-related quality of life (HRQoL), as measured by the 36-Item Short Form Health Survey (SF-36).

Methods

A cross-sectional study of 250 polytrauma patients in Islamabad was conducted using demographic data, a study-specific patient-reported questionnaire for delayed or unrecognized injuries (SSMIQ), and SF-36 scores. Statistical analyses included descriptive statistics, t-tests, ANOVA, Pearson correlation, and multiple regression using SPSS version 26.

Results

Most participants were male (n = 212, 85%) and aged 18-29 years (n = 120, 48%). Patient-reported missed or delayed injuries were identified in 191 (76%) patients and were associated with worse quality of life (r = -0.28, p = 0.001). Patients with reported missed or delayed injuries had lower SF-36 scores than those without such injuries (85.9 ± 6.2 vs. 88.5 ± 5.4, p = 0.015). Older age, male sex, ICU admission, surgery, and ambulance arrival were other factors associated with lower quality of life. An independent association with lower quality-of-life scores was observed for patient-reported missed or delayed injuries (B = -2.85, 95% CI: -4.26 to -1.44; p = 0.001).

Conclusion

Patient-reported missed or delayed injuries were associated with poorer HRQoL. An organized tertiary survey, thorough examination, and post-discharge follow-up may help reduce missed injuries and improve outcomes.

## Introduction

Polytrauma, characterized by multiple injuries involving organs or systems, is a leading cause of death among young adults and is associated with high morbidity and socioeconomic burden [1]. It is a multifaceted pathophysiological and immunological phenomenon that requires specific surgical strategies [2].

Missed injuries remain a common issue in polytrauma care, with reported incidence rates ranging from 1.3% to 39%. Among patients with missed injuries, approximately 15%-22.3% have clinically significant missed injuries [3]. Missed injuries may occur due to factors such as altered consciousness, hemodynamic instability, or diagnostic oversight, and comprehensive tertiary trauma surveys may help reduce the risk of missed injuries and their associated complications [4].

Transfers to other hospitals may increase the risk of delayed or missed injury detection, with researchers reporting that 35% of transferred trauma patients had one or more new diagnoses, more than half of which required management changes [5]. Despite advances in trauma care and standardized assessment protocols, clinically significant injuries may still be missed during primary and secondary trauma surveys, particularly in severely injured patients [6].

The SF-36 is a valid and valuable tool for measuring the physical and mental health of patients with physical and functional impairments. It has been reported to exhibit fewer ceiling effects than conventional disability scales [7]. Studies show a steady decline in health-related quality of life (HRQoL) following injury, and recovery does not necessarily restore the pre-injury level [8].

The severity of injury, pain, mental health, coping, and self-efficacy have been identified as factors that influence post-discharge HRQoL [9]. Older age, low socioeconomic status, and extremity injury are associated with poorer outcomes, and increased satisfaction with hospital care may positively influence recovery [10].

In Pakistan, the burden of trauma resulting from road traffic accidents, falls, and workplace injuries continues to rise. Polytrauma patients often sustain injuries involving multiple body regions, which can complicate the initial clinical assessment and increase the risk of missed or delayed diagnoses. However, there is little local evidence on the prevalence, patterns, and impact of missed injuries in polytrauma patients. Recognition of these trends is vital for advancing trauma care and improving patient outcomes, as undetected injuries can have wide-ranging physical, psychological, and social implications. This study aims to identify the prevalence and trends of missed injuries in polytrauma patients and to assess the correlation between missed injuries and patient-reported health outcomes measured by the SF-36 questionnaire. In addition, the study aims to evaluate the relationship between demographic factors, including age and sex, and patient-reported missed or delayed injuries.

## Materials and methods

The present cross-sectional study was conducted from September to December 2025 at a tertiary care hospital in Islamabad, Pakistan, to investigate the prevalence and trends of patient-reported missed or delayed injuries among polytrauma patients and their association with patient-reported health outcomes.

Sampling strategy and population size

The minimum required sample size was calculated as 385 participants using a 95% confidence level, a 5% margin of error, and an assumed prevalence of 50% due to the absence of local data [11]. A convenience sampling approach was used. During the study period, 385 polytrauma patients were approached for participation from hospitals in Islamabad. Eligible participants were adults aged ≥18 years with injuries involving at least two body regions who were clinically stable and able to provide informed consent. Patients with clinical instability, altered consciousness, cognitive impairment, incomplete questionnaire responses, or refusal to participate were excluded. After applying the eligibility criteria and excluding incomplete responses, 250 patients were included in the final analysis.

Data collection tools

The research utilized a structured questionnaire divided into three sections: demographic data, the Study-Specific Patient-Reported Missed Injuries Questionnaire (SSMIQ), and the SF-36 Health Survey. All questionnaires were administered in English during hospital admission or during follow-up visits after discharge, depending on the participants’ clinical stability. Those who could read and comprehend English completed the questionnaires independently. For participants with limited English comprehension and reading abilities, qualified research assistants clarified questions and read them aloud without interfering with their answers. This approach was used to improve understanding and ensure consistent data collection.

Demographic information

The first section included demographic and clinical variables, such as age, sex, marital status, education level, and occupation. The clinical variables included injury details, length of stay, and mechanism of trauma. This section aimed to provide information for subgroup analyses and to examine the relationship between demographic or clinical variables and the probability of patient-reported missed or delayed injuries.

SSMIQ

The second section was the SSMIQ, a study-specific instrument developed to identify possible patient-reported missed or delayed injuries among polytrauma patients. The SSMIQ was not a validated diagnostic tool; it was an exploratory screening tool used to collect patient-reported data on injuries that might otherwise have gone unnoticed during the initial clinical examination. Therefore, findings related to missed injuries in this study should be interpreted as patient-reported perceptions of missed or delayed injuries, rather than as clinically confirmed diagnostic errors. The present study did not include provider-led tertiary surveys, imaging review, or follow-up chart review to clinically validate missed injuries. The questionnaire was designed to include questions about anatomical areas, such as the head and face, spine, chest, abdomen, and extremities. Patients were asked whether they had sustained an injury in any of these areas that was either missed or diagnosed later during their treatment. Questions included examples such as: “Did you have a head or face injury that was not recognized during the initial assessment?” and “Was the injury diagnosed later during subsequent treatment or follow-up care?” Participants responded based on information provided by healthcare professionals during hospitalization or follow-up visits. This anatomically based methodology enabled the systematic identification of patient-reported missed or delayed injuries and the evaluation of their patterns and occurrence in the target population (Appendix 1).

Short Form-36 health survey (SF-36)

This study used the SF-36 Health Survey to measure quality of life (QoL) in polytrauma patients. The SF-36 is one of the most widely used tools for assessing HRQoL and was initially developed by Ware and Sherbourne in 1992. It comprises 36 items reflecting eight domains: physical functioning, role limitations due to physical health, bodily pain, general health perceptions, vitality, social functioning, role limitations due to emotional health, and mental health. All domains use a 0-100 scale, with higher scores indicating better health status and better QoL. Internal reliability has also been reported to be good, with Cronbach’s alpha values ranging from 0.78 to 0.93 across most domains. Its ability to assess both physical and psychological dimensions of well-being makes it especially applicable to polytrauma patients, as they often experience long-term effects from their injuries. It was used in its original form, the SF-36, which is freely available for research [12]. The development of the SF-36 is attributed to the RAND Corporation, and its implementation is outlined in their guidelines [13].

Analytical approach

IBM SPSS Statistics version 26 was used for data analysis. Descriptive statistics were used to summarize demographic and clinical variables. To test for normality, the Kolmogorov-Smirnov and Shapiro-Wilk tests were applied. The relationships between QoL and patient-reported missed or delayed injuries were examined using Pearson’s correlation, independent-samples t-tests, and one-way ANOVA with effect sizes, including partial eta-squared and Cohen’s d. Multiple linear regression was used to determine predictors of QoL. However, there were no formal injury severity indices, such as the Injury Severity Score (ISS), and ICU admission and surgery during hospitalization were used as proxy variables to partially explain injury severity in the regression analysis. The tests were conducted with a statistical significance level of 0.05. Variables were evaluated for multicollinearity using variance inflation factors, and no multicollinearity was found (VIF <2).

Ethical protocols

The Institutional Review Board of Riphah International University, IRB number Riphah/IRB/25/08/0253, pre-acknowledged the research. Verbal or written informed consent was obtained. Participants who struggled to comprehend English or had physical disabilities were allowed to complete the questionnaires independently or with the help of trained research assistants. Participation was voluntary, and the data were anonymized. The procedures were adjusted to be culturally sensitive and not overly burdensome to the participants.

## Results

Table 1 shows the demographic and clinical profiles of the study participants (N = 250). The majority were between 18 and 29 years old (n = 120, 48%), followed by 30-39 years (n = 55, 22%), 40-49 years (n = 40, 16%), 50-59 years (n = 25, 10%), and 60 years or above (n = 10, 4%). Most participants were men (n = 212, 85%), compared with women (n = 38, 15%). More than half were married (n = 135, 54%), while others were single (n = 66, 26%) or widowed/divorced (n = 49, 20%). Regarding educational status, most had primary or secondary education (n = 109, 44%), followed by higher secondary education (n = 87, 35%), no formal education (n = 40, 16%), and graduate/postgraduate qualification (n = 14, 6%). Occupation-wise, 108 (43%) were employed, 64 (26%) were unemployed, 61 (24%) were students, and 17 (7%) were retired. Falls from height were the most prevalent cause of injury (n = 91, 36%), followed by road traffic accidents (n = 63, 25%), assault (n = 58, 23%), crush injuries (n = 33, 13%), and other causes (n = 5, 2%). Almost half of the participants arrived on their own (n = 123, 49%), while the rest were transported by ambulance (n = 67, 27%) or other modes (n = 60, 24%). Most participants had a hospital stay of 1-2 weeks (n = 123, 49%). Sixty-two (25%) stayed for less than 1 week, and 65 (26%) stayed for 2 weeks or longer. ICU admission was required in 128 (51%) cases, while 122 (49%) did not require ICU admission. Surgery was performed in 144 patients (58%), whereas 106 (42%) did not undergo surgery. Concerning the time since injury, the largest proportion comprised patients injured 3-6 months earlier (n = 123, 49%), followed by less than 3 months (n = 79, 32%), 6-12 months (n = 40, 16%), and more than 12 months (n = 8, 3%). It is worth noting that the majority of participants (n = 191, 76%) had at least one missed injury, while only 59 (24%) had none.

**Table 1:** Demographic characteristics of participants (N = 250). Note: Values are presented as n (%). No statistical comparisons were performed for the demographic variables in this table. f: frequency; %: percentage.

Variable	n	%
Age		
18-29 years	120	48
30-39 years	55	22
40-49 years	40	16
50-59 years	25	10
≥60 years	10	4
Sex		
Male	212	85
Female	38	15
Marital status		
Single	66	26
Married	135	54
Widowed/divorced	49	20
Educational level		
No formal education	40	16
Primary/secondary	109	44
Higher secondary/intermediate/A-level	87	35
Graduate/postgraduate	14	6
Occupation		
Student	61	24
Employed	108	43
Unemployed	64	26
Retired	17	7
Mechanism of injury		
Road traffic accident	63	25
Fall from height	91	36
Assault	58	23
Crush injury	33	13
Other	5	2
Mode of arrival at the hospital		
Ambulance	67	27
Private transport	123	49
Other	60	24
Length of hospital stay		
<1 week	62	25
1-2 weeks	123	49
≥2 weeks	65	26
ICU admission		
Yes	128	51
No	122	49
Surgery required during admission		
Yes	144	58
No	106	42
Time since injury		
<3 months	79	32
3-6 months	123	49
6-12 months	40	16
>12 months	8	3
Missed vs. non-missed injury groups		
No missed injury	59	24
≥1 missed injury	191	76

Table 2 presents the correlation between the SSMIQ and the SF-36 among participants (N = 250). A moderate negative correlation was found (r = -0.28, t(248) = -4.59, p < 0.001), indicating that an increased score on the SSMIQ, which suggests a higher number of patient-reported missed or delayed injuries, was associated with a lower score on the SF-36, suggesting worse QoL.

**Table 2:** Pearson correlations between the SSMIQ and the SF-36 (N = 250). Note. Values represent Pearson correlation coefficients (r) between continuous variables. N = 250. Two-tailed p values <0.001 are denoted with double asterisks (**). SSMIQ: Study-Specific Patient-Reported Missed Injuries Questionnaire; SF-36: 36-Item Short Form Health Survey.

Variables	r	t	p
Study-Specific Patient-Reported Missed Injuries Questionnaire (SSMIQ) and 36-Item Short Form Health Survey (SF-36)	-0.28	-4.59	<0.001**

Table 3 compares the quality-of-life scores, measured using the SF-36, between patients with and without missed injuries. The mean SF-36 scores of those who had one or more missed injuries (M = 85.9, SD = 6.2) were lower than those of patients with no missed injuries (n = 59, 24%). This finding was statistically significant (t(248) = -2.45, p = 0.015), with a 95% CI ranging from -4.69 to -0.51. The effect size (Cohen’s d = 0.43) indicated a moderate difference, suggesting that missed injuries were associated with a significant decrease in QoL.

**Table 3:** Comparison of quality-of-life scores, measured using the SF-36, between patients with and without missed injuries (N = 250) Note. Values are presented as mean ± SD. Independent-samples t-tests were conducted to compare participants with and without missed injuries. Group sizes are shown as n (%). Reported statistics include p values, t values, 95% CIs, and effect sizes (Cohen’s d). A p-value <0.05 was considered statistically significant.

Variable	≥1 missed injury (n = 191; 76%) Mean ± SD	No missed injury (n = 59; 24%) Mean ± SD	t	p	95% CI LL	95% CI UL	Cohen’s d
36-Item Short Form Health Survey (SF-36)	85.9 ± 6.2	88.5 ± 5.4	-2.45	0.015*	-4.69	-0.51	0.43

Table 4 shows differences by age regarding patient-reported missed or delayed injuries and QoL (N = 250). The highest and lowest mean scores were observed in the ≥60 years group (M = 1.70, SD = 0.48) and the 50-59 years group (M = 1.10, SD = 0.31), respectively, with significant group differences, F(4,245) = 13.20, p < 0.001, η² = 0.18, indicating a considerable effect size. Regarding QoL, measured using the SF-36, the highest score was recorded in the youngest group, 18-29 years, with a mean score of 88.0 (SD = 5.5), and the lowest score was observed in the 60 years and above group (M = 81.0, SD = 6.5). This finding was also statistically significant, with F(4,245) = 4.80, p = 0.001, η² = 0.07, representing a moderate effect size. Overall, the older age group was more prone to missed injuries and had a lower QoL than younger cohorts.

**Table 4:** Age-group differences in patient-reported missed injuries and quality of life (N = 250). Note. Data are presented as mean ± SD; group sizes are shown as n (%). One-way ANOVA was conducted to examine differences across age groups. All comparisons were significant at p < 0.01. η² represents the eta-squared effect size.

Variable	18-29 years (n = 120; 48%)	30-39 years (n = 55; 22%)	40-49 years (n = 40; 16%)	50-59 years (n = 25; 10%)	≥60 years (n = 10; 4%)	F(4,245)	p-value	η²
Study-Specific Patient-Reported Missed Injuries Questionnaire (SSMIQ)	1.30 ± 0.46	1.25 ± 0.43	1.15 ± 0.36	1.10 ± 0.31	1.70 ± 0.48	13.2	<0.001	0.18
36-Item Short Form Health Survey (SF-36)	88.0 ± 5.5	86.5 ± 6.0	85.5 ± 5.8	84.0 ± 6.2	81.0 ± 6.5	4.8	0.001	0.07

Table 5 presents the findings of a multiple regression analysis examining predictors of QoL in 250 participants. Patient-reported missed or delayed injuries were a significant negative predictor (B = -2.85, 95% CI: -4.26 to -1.44, p < 0.001), indicating that patients with missed injuries reported lower QoL. Age (B = -0.98, 95% CI: -1.65 to -0.31, p = 0.004), male sex (B = -1.95, 95% CI: -3.70 to -0.20, p = 0.028), mode of hospital arrival (B = -1.25, 95% CI: -2.30 to -0.20, p = 0.015), ICU admission (B = -2.10, 95% CI: -3.60 to -0.60, p = 0.006), surgery during admission (B = -1.70, 95% CI: -3.10 to -0.30, p = 0.015), and time since injury (B = -0.88, 95% CI: -1.50 to -0.26, p = 0.007) were also significant predictors. All these findings indicate that patient-reported missed or delayed injuries, as well as demographic and clinical factors, were independently associated with lower quality-of-life scores.

**Table 5:** Multiple regression predicting quality of life using SF-36 scores from patient-reported missed injuries and demographic and clinical factors (N = 250). Note. R² = 0.32; adjusted R² = 0.30; F(8, 241) = 18.45. Multiple linear regression was conducted to identify predictors of quality-of-life scores measured using the SF-36. Values include unstandardized coefficients (B), 95% CIs, standard errors (SEs), standardized beta coefficients (β), and p-values. *p < 0.05, **p < 0.01, ***p < 0.001.

Predictor	B	SE	β	t	p	95% CI LL	95% CI UL
Constant	95.21	2.5	-	38.08	<0.001***	90.28	100.14
Missed injury group	-2.85	0.72	-0.21	-3.96	<0.001***	-4.26	-1.44
Age	-0.98	0.34	-0.165	-2.88	0.004**	-1.65	-0.31
Sex, male = 1	-1.95	0.88	-0.12	-2.22	0.028*	-3.68	-0.22
Mode of arrival at hospital	-1.25	0.51	-0.135	-2.45	0.015*	-2.26	-0.24
ICU admission	-2.1	0.76	-0.145	-2.76	0.006**	-3.59	-0.61
Surgery required during admission	-1.7	0.69	-0.14	-2.46	0.015*	-3.06	-0.34
Time since injury	-0.88	0.32	-0.125	-2.75	0.007**	-1.51	-0.25

## Discussion

In our study, participants commonly reported missed or delayed injuries, and these were significantly associated with poorer HRQoL. Previous literature has reported wide variation in the incidence of missed injuries among trauma patients, ranging from 1.3% to 39%, with approximately 15%-22% of missed injuries considered clinically significant [3]. Nevertheless, the current study was based on patient-reported perceptions of delayed or unrecognized injuries, unlike the aforementioned studies, which were grounded in objective confirmation by tertiary surveys, imaging, or chart review. Therefore, the findings should be interpreted as patient-reported experiences rather than clinically validated diagnostic errors. The higher prevalence observed in the present study could reflect broader patient experiences, such as delayed symptom identification, persistent pain, memory bias, or dissatisfaction with initial trauma treatment, rather than strictly clinically verified diagnostic oversight. Individuals with missed injuries scored lower on the SF-36, as previously reported; long-term impairment may be explained by chronic pain, disability, and psychosocial consequences [14].

Our research found that patient-reported missed or delayed injuries were more common among older patients, which may be attributable to frailty and comorbidities. Nonetheless, previous studies have reported that younger age is significantly associated with patient-reported missed or delayed injuries, suggesting that age-related susceptibility to these injuries may vary across populations and clinical settings [15]. Moreover, we found that older age and patient-reported missed or delayed injuries were associated with reduced QoL, suggesting a possible cumulative impact of aging and unrecognized injuries. Other studies have found similar outcomes in elderly populations, where exposure to trauma was strongly linked with worse HRQoL and life satisfaction [16].

In our regression analysis, among the predictors included in the model, patient-reported missed or delayed injuries exhibited the strongest independent negative association with SF-36 scores (B = -2.85, 95% CI: -4.26 to -1.44). Nonetheless, this association must be viewed with caution, given the study’s cross-sectional design, which precludes causal inference. This aligns with previous studies showing that polytraumatized patients develop chronic disabilities, pain, and psychological consequences years after trauma, leading to diminished long-term QoL [14]. Our findings indicated that older age was an independent factor associated with lower QoL, supporting previous research among older individuals that found that advanced age and exposure to traumatic events were associated with significantly lower HRQoL scores [16]. We found that male patients had substantially lower QoL than female patients. This is contrary to previous studies reporting that chronic pain was more prevalent in women and had a greater adverse effect on QoL [17]. One possible explanation is that, in developing settings such as Pakistan, male patients are more frequently exposed to high-impact trauma mechanisms, including road traffic accidents and occupational injuries, which may result in more severe physical and functional impairment. Variations in cultural, biological, and healthcare-seeking behaviors may also contribute to differences in QoL outcomes between males and females, warranting further investigation.

In addition, QoL scores were worse among patients transported to the hospital by ambulance, suggesting more serious injuries at presentation. Previous research has indicated that ambulance users perceive their situation as more severe and immediate than self-transport users do. Thus, mode of arrival may indicate underlying clinical severity; however, it is not necessarily an independent predictor of worse outcomes [18]. We found that ICU stay was associated with lower QoL scores, possibly reflecting more severe trauma. This finding aligns with a previous study that found that ICU patients experience numerous physical, social, and general health impairments several years after discharge, despite other patients recovering over time [19]. Patients in our study with a history of surgery scored lower on QoL, which may be explained by the physical and functional burden of their injuries and the obstacles they encountered during the healing process. This aligns with evidence suggesting that such surgical interventions, although potentially necessary, may affect QoL and mental health, which is why multifaceted postoperative care is considered essential [20]. Similarly, a longer time since injury was also associated with cumulative losses in QoL, again suggesting that long-term disability and related conditions may reduce life satisfaction [21]. Despite these considerations, the findings provide important insight into patient-reported experiences following polytrauma and highlight the potential impact of perceived missed or delayed injuries on long-term QoL.

Limitations and recommendations

This study has several limitations. First, the results may not apply to all trauma patients in Pakistan because a convenience sampling technique was used. Second, self-reported questionnaires were used to collect the data, which are prone to recall bias and underreporting. Additionally, the use of English-language questionnaires, even with assistance, may have introduced language-related response bias. Third, the study was conducted at a single tertiary care hospital in Islamabad, which may limit the generalizability of the findings to other regions with different healthcare resources, trauma systems, and clinical practices. Fourth, the cross-sectional design does not allow for the determination of causality between patient-reported missed or delayed injuries and long-term QoL outcomes. Additionally, standardized injury severity indices, anatomical injury burden, and baseline health status were not incorporated into the analysis, potentially leading to residual confounding. The questionnaire used to measure patient-reported missed or delayed injuries was a screening tool developed for this study, and its validity and reliability were not rigorously tested; therefore, it may not capture all clinically significant diagnostic oversights. Furthermore, patient-reported missed or delayed injuries were not independently confirmed by imaging review, tertiary trauma surveys, or chart review. They should therefore not be interpreted as clinically validated diagnostic errors. Therefore, the reported prevalence cannot be directly compared with estimates from research that uses clinically validated definitions of missed injuries.

These gaps should be addressed in future studies through multicenter prospective cohort studies with standardized diagnostic follow-up, including imaging and clinical re-examination. Extension to rural and resource-constrained settings would provide a more comprehensive view of patient-reported missed or delayed injuries across healthcare systems. Longitudinal studies are also necessary to track the long-term effects of patient-reported missed or delayed injuries on recovery pathways. Screening questionnaires should be validated and refined in future research to enable the proper detection of missed injuries. Lastly, evidence-based solutions to mitigate the burden of missed injuries could be explored through interventional research on strategies such as regular tertiary surveys, trauma team training using simulation, and post-discharge digital health interventions.

## Conclusions

Overall, this study suggests that patient-reported missed or delayed injuries, defined as injuries perceived by patients to have been identified after the initial trauma assessment, are prevalent in polytrauma patients and are significantly associated with lower HRQoL. Age, male sex, ICU admission, mode of hospital arrival, hospitalization, and surgery were also found to be significantly associated with lower QoL, with patient-reported missed or delayed injuries showing the largest association among the variables studied. These results underscore the importance of detailed clinical examination, systematic tertiary surveys, and close follow-up in trauma management. Addressing diagnostic gaps is essential, as this may improve long-term functional recovery, rehabilitation outcomes, and overall patient well-being.

## Appendices

Appendix 1 

**Table 6:** Study-Specific Patient-Reported Missed Injuries Questionnaire (SSMIQ).

Body region	Missed injury identified later
Head and face	☐ Yes ☐ No
Neck	☐ Yes ☐ No
Chest	☐ Yes ☐ No
Abdomen and pelvis	☐ Yes ☐ No
Extremities (arms/legs)	☐ Yes ☐ No
Spine (back/neck bones)	☐ Yes ☐ No
